# Trabecular Meshwork Thickness Measured by Swept-Source AS-OCT as a Predictor of Surgical Outcomes After Trabecular Micro-Bypass Stent Implantation

**DOI:** 10.3390/jcm15093341

**Published:** 2026-04-27

**Authors:** Heejin Yoon, Jiwoong Lee, Seung Min Lee, Ji Eun Lee, Su Jin Kim, Sangwoo Moon

**Affiliations:** 1 Department of Ophthalmology, Pusan National University Yangsan Hospital, Pusan National University School of Medicine, Yangsan 50612, Republic of Korea; gmlwls1447@naver.com (H.Y.); platinummetal@hanmail.net (S.M.L.); jiel75@pusan.ac.kr (J.E.L.); pearlsj@hanmail.net (S.J.K.); 2 Research Institute for Convergence of Biomedical Science and Technology, Pusan National University Yangsan Hospital, Yangsan 50612, Republic of Korea; 3 Department of Ophthalmology, Pusan National University Hospital, Pusan National University School of Medicine, Busan 49241, Republic of Korea; glaucoma@pusan.ac.kr

**Keywords:** open-angle glaucoma, trabecular micro-bypass stent, minimally invasive glaucoma surgery, trabecular meshwork, anterior segment swept-source optical coherence tomography

## Abstract

**Background:** Although minimally invasive glaucoma surgery (MIGS), including trabecular micro-bypass stent implantation, is safe, its surgical outcomes remain variable, with limited reliable success predictors. We evaluated whether preoperative trabecular meshwork (TM) thickness is associated with surgical outcomes after iStent inject^®^ W implantation. **Methods:** Patients with open-angle glaucoma (*n* = 28) who underwent iStent inject^®^ W implantation were included. Preoperative AS-SS-OCT was used to measure TM thickness and conventional angle parameters, including angle opening distance, angle recess area, trabecular–iris space area, and trabecular–iris angle. Surgical success was IOP ≤ 15 mmHg with ≥25% reduction or final IOP ≤ 12 mmHg under specified conditions. Logistic regression analyses were performed to identify factors associated with surgical outcomes. **Results**: At 12 months, 22 eyes (78.6%) achieved surgical success. Mean TM thickness was significantly greater in the unsuccessful than in the successful group (250.62 ± 32.05 μm vs. 180.75 ± 30.61 μm, *p* = 0.001), with similar findings for nasal and temporal TM thickness. Conventional angle parameters were not associated with surgical outcomes. In univariable analysis, both mean and nasal TM thickness were significantly associated with an increased risk of failure (per 10 μm increase; mean TM: OR = 2.77, 95% CI = 1.12–6.86, *p* = 0.027; nasal TM: OR = 1.64, 95% CI = 1.04–2.58, *p* = 0.034). **Conclusions**: Increased preoperative mean and nasal TM thickness was significantly associated with surgical failure following iStent inject^®^ W implantation. TM’s microstructural properties are more relevant than angular configuration in determining MIGS outcomes. Preoperative assessment of TM thickness using AS-SS-OCT may serve as a useful imaging biomarker for optimizing patient selection.

## 1. Introduction

Minimally invasive glaucoma surgery (MIGS) has emerged as an increasingly adopted surgical option for the management of glaucoma, offering a favorable safety profile and rapid postoperative recovery compared to traditional filtering surgeries [1,2]. Among these techniques, trabecular bypass procedures, such as iStent implantation, enhance aqueous humor outflow by bypassing the trabecular meshwork (TM), thereby lowering the intraocular pressure (IOP) [3,4]. Despite these advantages, the IOP-lowering effect of MIGS remains variable, and a subset of patients experience suboptimal outcomes or require additional glaucoma surgery [4,5]. Identifying reliable preoperative predictors of surgical success is essential to optimizing patient selection and improving clinical outcomes.

Previous studies have explored different clinical factors associated with MIGS outcomes, including preoperative IOP, glaucoma severity, and medication burden [3,4,5,6,7]. Although some of these factors have demonstrated partial associations with surgical success, the results have been inconsistent, and their predictive value remains limited [3,4,5,6,7]. Importantly, these conventional parameters do not directly reflect the functional status of the aqueous outflow pathway, which is the primary target of trabecular bypass procedures.

The TM plays a central role in regulating the aqueous humor outflow and is considered the main pathological site of outflow resistance in glaucomatous eyes [8,9]. Structural and histopathological changes in the TM, such as increased extracellular matrix deposition, fibrosis, and cellular alterations, have been implicated in elevated outflow resistance and impaired aqueous drainage [10,11]. Given that trabecular bypass procedures rely on the integrity and function of the distal outflow system beyond the TM, the intrinsic structural characteristics of the TM may influence the surgical efficacy. However, the relationship between TM morphology and MIGS outcome has not been comprehensively elucidated.

Anterior segment optical coherence tomography (AS-OCT) is a non-invasive and reproducible method for visualizing anterior chamber angle structures [12,13,14,15]. Previous AS-OCT studies have primarily focused on angle configuration parameters, such as angle opening distance (AOD), trabecular–iris space area (TISA), and trabecular–iris angle (TIA), to assess angle anatomy [16,17]. However, these parameters mainly reflect the geometric configuration of the anterior chamber angle and do not capture the microstructural properties of the TM. Recent advances in high-resolution imaging have enabled a more detailed visualization and quantitative assessment of TM structures, offering a potential imaging biomarker that may better reflect the structural status of the outflow pathway [13,14,15].

In this context, we investigated whether preoperative TM thickness, measured using anterior segment swept-source OCT, is associated with surgical outcomes following trabecular micro-bypass stent (iStent inject^®^ W) implantation. Furthermore, we evaluated the clinical utility of TM thickness as a practical imaging biomarker for preoperative risk stratification and surgical decision-making in a real-world setting. In addition, we evaluated the relationship between the conventional angle parameters and surgical success to determine their comparative predictive value.

## 2. Materials and Methods

### 2.1. Ethics Statement

This single-center retrospective study was approved by the Institutional Review Board of Pusan National University Yangsan Hospital (approval no. 55-2024-085). All patients provided written informed consent for the surgical procedures and their data to be stored in the hospital database and used for research. This study was conducted in accordance with the principles of the Declaration of Helsinki.

### 2.2. Study Design

This retrospective cohort study included 28 eyes of 28 patients who underwent iStent inject^®^ W implantation between January 2018 and May 2024, either alone or in combination with phacoemulsification, for the management of open-angle glaucoma at the Department of Ophthalmology at Pusan National University Yangsan Hospital (Yangsan, Korea). All patients were followed up for at least 12 months postoperatively. When both eyes underwent an iStent inject^®^ W, only the first operated eye was included in the analysis. OAG was diagnosed based on the presence of glaucomatous optic disc changes and corresponding visual field defects as confirmed by two reliable visual field tests and an open anterior chamber angle [18]. Patients with a history of ocular surgery other than uncomplicated cataract extraction were excluded. Other exclusion criteria were eyes with coexisting neurological or retinal diseases capable of affecting the visual field or optic nerve status.

Baseline demographic and clinical data, including age, sex, glaucoma diagnosis, preoperative IOP (measured at the time of AS-OCT), visual acuity, number and duration of glaucoma medications, lens status, central corneal thickness, axial length, and spherical equivalents, were collected. Structural and functional glaucoma severity was assessed using the circumpapillary retinal nerve fiber layer (cpRNFL) thickness, macular ganglion cell/inner plexiform layer (mGC/IPL) thickness, and standard automated perimetry parameters. Optic disc stereophotographs were obtained, and ocular biometry was performed using IOL Master (Carl Zeiss Meditec; Dublin, CA, USA). Standard automated perimetry was conducted using a Humphrey Visual Field Analyzer 3 (Model 800; Carl Zeiss Meditec, Dublin, CA, USA) with the Swedish Interactive Threshold Algorithm 24-2 program. Retinal nerve fiber layer (RNFL) and ganglion cell–inner plexiform layer thicknesses were measured using Cirrus High-Definition Optical Coherence Tomography 5000 (Carl Zeiss Meditec; Dublin, CA, USA). Central corneal thickness was measured using an ultrasonic pachymeter (Pachmate; DGH Technology Inc., Exton, PA, USA), and autorefraction was performed using a Canon RF-F2 (Canon Inc., Tokyo, Japan).

### 2.3. Surgical Technique

All surgeries were performed under local anesthesia by a single glaucoma specialist (S. M.). Following standard cataract surgery (if needed), trabecular micro-bypass stents (iStent inject^®^ W) were introduced. The iStent inject^®^ W device contains two preloaded, heparin-coated titanium stents designed for single use. Each stent was placed *ab interno* through the nasal TM into the SC using a Swan-Jacob gonioprism, with the second stent positioned approximately 2–3 clock hours apart from the first. After implantation, the ophthalmic viscoelastic device was removed, and the anterior chamber was refilled with a balanced salt solution.

Postoperatively, patients received topical moxifloxacin (Moroxacin^®^, Hanmi Pharm, Co., Seoul, Korea) four times daily and fluorometholone (Flumetholone^®^, Santen Pharmaceutical, Osaka, Japan) four times daily for 1 week, followed by gradual tapering over 4 weeks.

### 2.4. Indications for Surgery and Definition of Success

Surgical indications included (1) inadequate IOP control despite maximally tolerated medical therapy, (2) intolerance or allergy to glaucoma medications, and (3) patient agreement to undergo immediate additional glaucoma surgery (including trabeculectomy or tube shunt surgery), if required.

Patients were divided into two groups according to surgical outcomes at 12 months: successful and unsuccessful. Qualified surgical success was defined as achieving an IOP ≤ 15 mmHg with glaucoma medications and meeting one of the following criteria: IOP reduction ≥ 25% or final IOP ≤ 12 mmHg if baseline IOP was ≤15 mmHg [19,20]. Surgical failure was defined as follows: (1) not meeting the above criteria at two consecutive visits 1 month postoperatively, (2) loss of light perception, and (3) need for additional glaucoma surgery, defined as trabeculectomy or tube shunt implantation [19].

### 2.5. AS SS-OCT Imaging and Measurements

All patients underwent preoperative anterior segment swept-source optical coherence tomography (AS SS-OCT) using CASIA2 (Tomey Corporation; Nagoya, Japan), which utilizes a 1310-nm swept-source laser wavelength and a scanning speed of 50,000 A-scans per second. The device had a penetration depth of up to 14 mm, a transverse resolution of ≤30 μm, and an axial resolution of ≤10 μm. The structural parameters were measured using the device’s built-in software (version: 4B.2).

**Figure 1:**
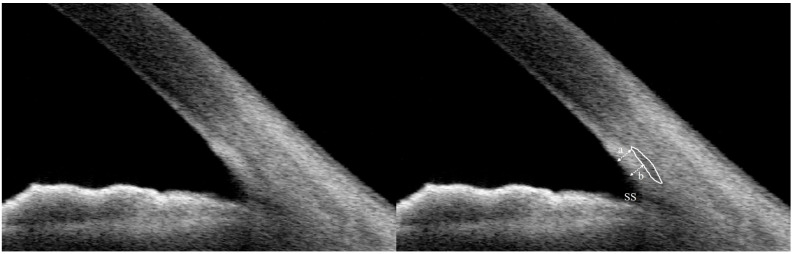
Anterior segment swept-source optical coherence tomography image for measuring trabecular meshwork thickness. The *white oval line* was identified as SC, and its reflectivity was lower than that of its adjacent tissues. The thickness of TM was calculated as the average of two measurements, the perpendicular distance from the anterior endpoint (a) and midpoint (b) of SC to the inner layer of the TM, indicated by the *dashed arrows* TM, trabecular meshwork; SC, Schlemm’s canal; SS, scleral spur.

Angle configuration parameters, including angle opening distance (AOD), angle recess area (ARA), trabecular–iris space area (TISA), and trabecular–iris angle (TIA), were measured at 500 μm and 750 μm from the scleral spur. TM thickness was measured as previously described. Briefly, Schlemm’s canal (SC) was identified as a low-reflectivity, oval, or slit-like structure adjacent to the scleral spur. The TM thickness was defined as the average of two perpendicular measurements from the anterior endpoint and midpoint of the SC to the inner boundary of the TM (Figure 1) [21].

### 2.6. Statistical Analysis

IBM SPSS ver. 26.0 (IBM Corp., Armonk, NY, USA) was used for all statistical analyses. The normality of numerical data distributions was assessed using the Kolmogorov–Smirnov test. Continuous variables are presented as mean ± standard deviation, and categorical variables as counts and percentages. The Snellen visual acuity was converted to the logarithm of the minimal angle of resolution (logMAR) equivalents for data analysis. Interobserver and intraobserver reproducibility of TM thickness measurements were assessed using intraclass correlation coefficients (ICCs). A second masked observer (H.Y) independently performed measurements for interobserver analysis, while repeated measurements by a single observer (S.M) at two separate time points (>2 months apart) were used for intraobserver analysis. ICCs were calculated using two-way random-effects and two-way mixed-effects models, respectively, based on absolute agreement. Univariate logistic regression analyses were performed to identify factors associated with surgical success. Variables with potential clinical relevance or statistical significance were included in multivariate logistic regression models. Because of the limited number of events, separate multivariate models were constructed to avoid overfitting. Each model included the axial length, preoperative IOP, and one TM thickness parameter (mean, nasal, or temporal TM thickness). Variables were scaled to represent the odds ratio (OR) per 10 μm increase in TM thickness. Diagnostic performance was evaluated using Receiver operating characteristic (ROC) curve analysis, and the optimal cut-off values were determined by the Youden index. The spherical equivalent was not included in the multivariate models because of its potential susceptibility to lenticular changes, particularly in eyes that underwent combined cataract surgery. A *p*-value < 0.05 was considered statistically significant.

## 3. Results

### 3.1. Demographics and Clinical Characteristics in All Patients

A total of 28 eyes were included, of which 22 (78.6%) achieved successful IOP control at 12 months, and 6 (21.4%) were classified as unsuccessful. Among them, 23 eyes underwent combined phacoemulsification with iStent implantation, and 5 eyes underwent standalone iStent implantation. Eighteen patients (64.3%) were diagnosed with primary OAG and 10 (35.7%) with pseudoexfoliation glaucoma. One eye was pseudophakic at the time of the surgery. Three eyes in the unsuccessful group required a trabeculectomy. Table 1 presents baseline clinical characteristics and patient demographics.

No significant differences were present between the groups in age, sex, glaucoma type, surgical method, visual acuity, number or duration of glaucoma medications, central corneal thickness, axial length, or glaucoma severity. However, preoperative IOP was significantly higher in the unsuccessful group compared to the successful group (27.00 ± 6.26 mmHg vs. 19.77 ± 5.14 mmHg, *p* = 0.005). In addition, the spherical equivalent was significantly more myopic in the unsuccessful group (*p* = 0.024), whereas axial length did not differ significantly between the groups. The number of glaucoma medications decreased from 3.82 ± 0.55 preoperatively to 3.54 ± 0.96 at 12 months, reaching statistical significance (*p* = 0.031), although the magnitude of reduction was modest.

### 3.2. AS SS-OCT Parameters and Surgical Outcomes

Among the anterior segment parameters, TM thickness displayed significant differences between the groups (Table 2).

The mean TM thickness was significantly greater in the unsuccessful group compared to the successful group (250.62 ± 32.05 μm vs. 180.75 ± 30.61 μm, *p* = 0.001). Similarly, both the nasal and temporal TM thicknesses were significantly greater in the unsuccessful group (*p* = 0.012 and *p* < 0.001, respectively). All measured TM thickness parameters, including the mean, nasal, and temporal values, were consistently greater in the unsuccessful group. In contrast, conventional angle parameters, including AOD, ARA, TISA, and TIA at both 500 μm and 750 μm, were not significantly associated with surgical outcomes.

### 3.3. Factors Associated with Surgical Success

The univariate logistic regression analysis revealed that axial length, preoperative IOP, mean TM thickness, and nasal TM thickness were significantly associated with surgical success. The odds ratios are presented per 10 µm increase in TM thickness. In univariable logistic regression analysis, mean TM thickness (per 10 μm increase; OR 2.77, 95% CI 1.12–6.86, *p* = 0.027) and nasal TM thickness (per 10 μm increase; OR 1.64, 95% CI 1.04–2.60, *p* = 0.034) were significantly associated with surgical failure. Temporal TM thickness showed a similar trend (per 10 μm increase; OR 3.73, 95% CI 0.86–16.18), but did not reach statistical significance (*p* = 0.079) (Table 3).

In multivariable analysis, mean and nasal TM thickness were included as potential predictors of surgical failure (Supplementary Table S1). ROC analysis demonstrated that mean TM thickness showed excellent discriminative ability for predicting surgical failure, with an area under the curve (AUC) of 0.955 (95% CI, 0.86–1.00). The optimal cut-off value was 211.9 μm, yielding a sensitivity of 100% and a specificity of 86.4%. However, these findings should be interpreted with caution given the small sample size and limited number of failure events.

### 3.4. Reproducibility of Trabecular Meshwork Thickness Measurements

The reproducibility of TM thickness measurements was good to excellent. Interobserver Intraclass Correlation Coefficients (ICC) values were 0.831 (95% CI, 0.640–0.928) for mean TM thickness, 0.838 (95% CI, 0.658–0.931) for temporal TM thickness, and 0.853 (95% CI, 0.692–0.939) for nasal TM thickness. Intraobserver ICC values were 0.940 (95% CI, 0.861–0.976), 0.928 (95% CI, 0.827–0.971), and 0.929 (95% CI, 0.832–0.971), respectively.

## 4. Discussion

This study investigated the association between preoperative anterior segment parameters and surgical outcomes after trabecular micro-bypass stent (iStent inject^®^ W) implantation. The principal finding was that greater TM thickness, as measured using anterior segment swept-source optical coherence tomography, was significantly associated with surgical failure. In univariable analysis, both mean and nasal TM thickness were significantly associated with an increased risk of failure, while temporal TM thickness showed a similar trend but did not reach statistical significance. These findings suggest that TM thickness may serve as a potential imaging biomarker for predicting surgical outcomes [10]. However, given the small sample size and limited number of failure events, the results should be interpreted as exploratory and hypothesis-generating.

Variability in surgical outcomes following MIGS has been increasingly recognized in clinical practice [1,2,3,4,22]. Although trabecular bypass procedures, such as iStent, are designed to enhance the aqueous humor outflow by bypassing the TM, their efficacy is inherently dependent on the patency and function of the distal outflow system, including SC and collector channels [8,10,11,23]. In this context, our findings suggest that the intrinsic structural characteristics of the TM play a critical role in determining surgical success. A thicker TM may reflect underlying pathological changes, such as increased extracellular matrix deposition, fibrosis, or cellular dysfunction, which are known contributors to increased outflow resistance in glaucomatous eyes [7,8,10,11,24]. These structural abnormalities may persist despite TM bypass, thereby limiting the effectiveness of trabecular bypass procedures [23]. The characteristics of the failure group observed in our study are consistent with previous reports suggesting that suboptimal outcomes following iStent implantation are associated with increased outflow resistance rather than angle configuration alone.

The present findings may be explained by the fundamental role of the TM in regulating the aqueous humor outflow resistance. According to the current understanding, the primary site of resistance is located at the juxtacanalicular tissue and the inner wall of Schlemm’s canal, where complex interactions between extracellular matrix components, tissue geometry, and endothelial pore formation determine the outflow facility [8,9,10,11,25]. Structural alterations in this region, including increased tissue thickness or stiffness, may therefore increase hydraulic resistance and limit aqueous outflow [8,9,10,11,25]. In this context, the observed association between increased TM thickness and poorer surgical outcomes may reflect underlying structural resistance that is not overcome by trabecular bypass procedures alone.

In addition, the aqueous humor outflow was segmental and heterogeneous, with regions of high- and low-flow distributed along the trabecular meshwork [25]. This segmental nature implies that localized structural changes may disproportionately influence the overall outflow resistance [25]. Furthermore, even when proximal resistance at the TM is bypassed, residual resistance in distal pathways, including the collector channels and deep scleral plexus, may persist and limit intraocular pressure reduction [25]. These considerations may explain why conventional angle parameters were not associated with surgical outcomes in the present study, whereas TM thickness, which directly reflects the structural properties of the outflow pathway, demonstrated a significant relationship with surgical success.

Interestingly, conventional angle-configuration parameters, including AOD, TISA, TIA, and ARA, were not significantly associated with surgical outcomes in our study. These findings suggest that geometric openness of the anterior chamber angle alone may not adequately reflect the functional status of the aqueous outflow pathway [9,10,11,12]. Although the angle width is important for surgical accessibility, it does not necessarily correspond to the resistance within the trabecular or post-trabecular outflow system. Therefore, the microstructural properties of the TM, which are not captured by traditional angle parameters, may be more relevant in determining the postoperative IOP control [8,9,10,11,16,17,25]. This distinction has important clinical implications, as it highlights the limitations of relying solely on angle configuration during preoperative evaluation and supports the role of TM-specific imaging biomarkers [9,12].

Consistent with previous studies, preoperative IOP has been demonstrated to influence surgical outcomes after MIGS [5,26,27]. Eyes with an elevated baseline IOP may represent a more advanced or treatment-resistant disease, potentially involving greater dysfunction of the distal outflow system [5,26,27]. Although axial length was associated with surgical outcomes in the univariate analysis, its clinical relevance remains uncertain. Notably, the spherical equivalent differed between the groups, whereas the axial length did not, suggesting that refractive differences may be influenced by lenticular factors rather than by true axial elongation [28]. Given that most patients underwent combined cataract surgery, the spherical equivalent may not accurately reflect intrinsic ocular structural characteristics in this setting and was therefore excluded from the multivariate analysis [28].

The use of swept-source AS-OCT enabled detailed visualization and quantitative assessment of the TM, providing a noninvasive method to evaluate the structural features of the outflow pathway [29]. Compared to conventional imaging parameters that primarily assess angle configuration, TM thickness may serve as a surrogate marker for tissue-level changes that influence aqueous outflow resistance [16,17,29,30]. From a clinical perspective, this imaging-based biomarker may facilitate preoperative risk stratification and improve patient selection for trabecular bypass. Particularly, patients with increased TM thickness may have a lower likelihood of achieving adequate IOP reduction and may be better candidates for alternative surgical approaches. However, TM thickness represents only one component of the aqueous outflow pathway and does not fully reflect downstream resistance including SC and collector channels, which may also contribute to surgical outcomes.

An important consideration in the present study was the selection of TM thickness as the primary structural parameter. Although SC can be visualized using AS-SS-OCT, its morphology is often variable, and its outer wall may be indistinct or difficult to delineate in a significant proportion of eyes [13]. This limitation can reduce measurement reproducibility and restrict its utility in routine clinical practice [13]. In contrast, TM typically exhibits relatively well-defined boundaries, allowing for more consistent and reproducible quantification [29,30]. TM thickness measurements demonstrated good interobserver agreement and excellent intraobserver reproducibility, with narrow confidence intervals, supporting the robustness of this parameter as a clinically applicable imaging biomarker. Therefore, TM thickness may represent a more robust and clinically feasible imaging biomarker, particularly in real-world settings where image quality and anatomical variability are inevitable [13,29,30].

This study has several limitations. First, the sample size was relatively small, particularly in the unsuccessful group, which may have increased the risk of statistical overfitting and limited the generalizability of the findings. Therefore, these results should be interpreted with caution. In addition, although temporal TM thickness differed significantly between groups, it did not reach statistical significance in regression analysis. This discrepancy may reflect the limited number of failure events and the resulting instability of the regression model. Accordingly, multivariable analyses should be considered exploratory. Although separate multivariate models have been constructed to mitigate this issue, larger studies are required for their validation. Nevertheless, this study serves as a clinical pilot investigation, providing preliminary but compelling evidence that the TM microstructure is a key determinant of iStent success. To build upon these initial findings, future prospective studies with larger multicenter cohorts and longitudinal follow-up are warranted to establish definitive diagnostic thresholds for TM thickness. Furthermore, integrating advanced functional imaging techniques, such as phase-sensitive OCT to assess TM motion, could provide a more comprehensive understanding of the relationship between TM structural stiffness and distal outflow resistance. Second, the retrospective design may have introduced selection bias. Third, although standardized measurement protocols were used, TM thickness measurements may have been influenced by image quality and observer variability [13,29,30]. Finally, although TM thickness was associated with surgical outcomes, it did not comprehensively capture the functional status of the distal outflow system, which also plays a critical role in determining surgical efficacy [15].

Despite these limitations, this study provided clinically meaningful insights into the structural determinants of MIGS outcomes. To the best of our knowledge, a few studies have specifically evaluated TM thickness as a predictor of surgical success using AS-OCT. Future prospective studies with larger sample sizes and longitudinal follow-up are warranted to validate these findings and further explore the integration of TM imaging into clinical decision-making.

## 5. Conclusions

Increased TM thickness was significantly associated with surgical failure following trabecular micro-bypass stent (iStent inject^®^ W) implantation, whereas conventional angle parameters were not predictive of outcomes. These findings suggest that the structural characteristics of TM, rather than angle configuration alone, may be potential predictors of MIGS success. From a clinical perspective, the preoperative assessment of TM thickness using AS-SS-OCT may provide a practical and noninvasive tool for risk stratification and patient selection in routine practice. Incorporating TM thickness into preoperative evaluation may help identify patients who are less likely to benefit from trabecular bypass procedures and guide the consideration of alternative surgical strategies.

## Figures and Tables

**Table 1:** Demographics and clinical characteristics of patients.

	Entire	Successful	Unsuccessful	*p*-Value *
Number of eyes (patients)	28 (28)	22 (22)	6 (6)	
Age at iStent, years	60.75 ± 17.94 (22.0–80.1)	60.92 ± 18.14 (22.0–80.1)	60.14 ± 18.87 (26.1–75.3)	>0.999
Sex, female	15 (53.6)	11 (50.0)	4 (66.7)	0.655
Diagnosis				>0.999
Primary open angle glaucoma	18	14	4	
Pseudoexfoliation glaucoma	10	8	2	
Surgical methods				0.285
iStent alone	5	3	2	
iStent combined phacoemulsification	23	19	4	
Successful IOP control at 1 year, %	22 (78.6)			
Additional glaucoma surgery	3 (13.6)	0	3 (Trabeculectomy)	
Preoperative IOP, mmHg, mmHg (at the time of AS-OCT test)	21.32 ± 6.08 (13–38)	19.77 ± 5.14 (13–37)	27.00 ± 6.26 (22–38)	0.005
Preoperative visual acuity, logMAR	0.43 ± 0.58 (3.0–0)	0.43 ± 0.62 (3.0–0)	0.43 ± 0.41 (1.0–0)	0.682
Number of glaucoma medication	3.82 ± 0.55 (2–4)	3.77 ± 0.61 (2–4)	4.00 ± 0.00 (4)	0.643
History of glaucoma medication, year	5.18 ± 5.89	5.15 ± 6.34	5.23 ± 4.31	0.494
Preoperative lens status, phakic	27 (96.4)	22 (100.0)	5 (83.3)	0.214
Central corneal thickness, μm	516.00 ± 32.98	511.96 ± 34.17	530.83 ± 25.22	0.194
Axial length, mm	24.71 ± 2.15	24.19 ± 0.88	26.62 ± 4.00	0.395
Spherical equivalent, diopters	–2.43 ± 4.66	–1.10 ± 2.16	–7.30± 7.82	0.024
cpRNFL thickness, μm	64.22 ± 11.00	63.43 ± 12.05	67.00 ± 6.00	0.216
mGC/IPL thickness, μm	59.12 ± 10.90	60.86 ± 10.21	54.83 ± 12.94	0.512
Visual field parameter				
Visual Field Index, %	53.93 ± 29.24	50.52 ± 30.75	65.83 ± 21.09	0.345
Mean deviation, dB	–16.55 ± 8.20	–17.37 ± 8.64	–13.69 ± 6.20	0.345
Pattern standard deviation, dB	8.79 ± 3.41	8.72 ± 3.62	9.01 ± 2.79	0.887

Counting fingers at 30 cm was considered equivalent to a Snellen value of 20/2000, which corresponded to a logMAR value of 2.0. Hand motion acuity was considered equivalent to a Snellen value of 20/20,000, which corresponded to a logMAR value of 3.0. Values are presented as mean ± standard deviation (range) or number (%) unless otherwise indicated. * Comparison between successful and unsuccessful eyes. AS-OCT, anterior segment optical coherence tomography; cpRNFL, circumpapillary retinal nerve fiber layer; IOP, intraocular pressure; logMAR, logarithm of the minimum angle of resolution; mGC/IPL, macular ganglion cell/inner plexiform layer.

**Table 2:** Preoperative anterior chamber angle parameters assessed with anterior-segment optical coherence tomography according to surgical success at 12 months.

Angle Parameters	Entire, *n* = 28	Successful, *n* = 22	Unsuccessful, *n* = 6	*p*-Value *
Mean TM thickness, μm	195.72 ± 42.09	180.75 ± 30.61	250.62 ± 32.05	0.001
Nasal TM thickness, μm	182.93 ± 43.35	170.94 ± 33.02	226.89 ± 51.13	0.012
500 AOD, mm	0.47 ± 0.22	0.49 ± 0.24	0.42 ± 0.15	0.780
500 ARA, mm^2^	0.16 ± 0.08	0.16 ± 0.08	0.13 ± 0.04	0.576
500 TISA, mm^2^	0.15 ± 0.07	0.16 ± 0.08	0.13 ± 0.04	0.538
500 TIA, °	42.82 ± 13.66	44.42 ± 14.53	36.98 ± 8.30	0.401
750 AOD, mm	0.68 ± 0.26	0.69 ± 0.28	0.63 ± 0.16	0.576
750 ARA, mm^2^	0.31 ± 0.14	0.32 ± 0.15	0.27 ± 0.08	0.779
750 TISA, mm^2^	0.30 ± 0.13	0.31 ± 0.14	0.26 ± 0.08	0.737
750 TIA, °	44.39 ± 17.33	46.77 ± 18.18	35.65 ± 10.86	0.218
Temporal TM thickness, μm	208.52 ± 46.92	190.57 ± 34.68	274.35 ± 14.81	<0.001
500 AOD, mm	0.52 ± 0.25	0.55 ± 0.26	0.44 ± 0.21	0.370
500 ARA, mm^2^	0.17 ± 0.08	0.19 ± 0.08	0.12 ± 0.05	0.104
500 TISA, mm^2^	0.17 ± 0.08	0.18 ± 0.08	0.12 ± 0.05	0.179
500 TIA, °	48.47 ± 16.97	49.81 ± 17.62	43.59 ± 14.69	0.417
750 AOD, mm	0.76 ± 0.33	0.78 ± 0.34	0.65 ± 0.31	0.263
750 ARA, mm^2^	0.34 ± 0.15	0.36 ± 0.16	0.26 ± 0.12	0.218
750 TISA, mm^2^	0.33 ± 0.15	0.35 ± 0.15	0.26 ± 0.11	0.218
750 TIA, °	46.31 ± 14.91	47.57 ± 15.38	41.66 ± 13.24	0.341

Values are presented as mean ± standard deviation. * Comparison between successful and unsuccessful eyes. AOD, angle opening distance; ARA, angle recess area; TIA, trabecular iris angle; TISA, trabecular iris space area; TM, trabecular meshwork.

**Table 3:** Preoperative variables associated with successful IOP control determined with step-wise logistic regression analyses.

	Univariate	
Parameters	OR (95% CI)	*p*-Value
Sex (reference male)	2.000 (0.302, 13.265)	0.473
Age	0.998 (0.948, 1.049)	0.923
Glaucoma type (reference POAG)	0.875 (0.130, 5.890)	0.891
Axial length	1.642 (1.017, 2.651)	0.042
Spherical equivalent	0.706 (0.479, 1.040)	0.078
Preoperative visual acuity (logMAR)	1.019 (0.210, 4.948)	0.981
Preoperative cpRNFL thickness	1.030 (0.950, 1.117)	0.480
Preoperative mGC/IPL thickness	0.947 (0.864, 1.037)	0.241
Preoperative Visual Field Index	1.020 (0.985, 1.056)	0.261
Preoperative mean deviation	1.062 (0.940, 1.200)	0.332
Preoperative IOP at AS-OCT test	1.226 (1.015, 1.482)	0.035
Angle parameters using AS-OCT		
Mean TM thickness, per 10 μm	2.772 (1.120, 6.860)	0.027
Nasal TM thickness, per 10 μm	1.642 (1.038, 2.579)	0.034
500 AOD, mm	0.174 (0.001, 22.500)	0.481
500 ARA, mm^2^	0.002 (<0.001, 3090.686)	0.389
500 TISA, mm^2^	<0.001 (<0.001, 2345.283)	0.323
500 TIA, °	0.950 (0.871, 1.036)	0.244
750 AOD, mm	0.321 (0.007, 15.166)	0.563
750 ARA, mm^2^	0.066 (<0.001, 150.053)	0.491
750 TISA, mm^2^	0.044 (<0.001, 140.267)	0.447
750 TIA, °	0.947 (0.874, 1.026)	0.181
Temp TM thickness, per 10 μm	3.728 (0.859, 16.179)	0.079
500 AOD, mm	0.141 (0.002, 9.578)	0.363
500 ARA, mm^2^	<0.001 (<0.001, 55.322)	0.121
500 TISA, mm^2^	<0.001 (<0.001, 63.431)	0.126
500 TIA, °	0.977 (0.923, 1.034)	0.424
750 AOD, mm	0.268 (0.013, 5.691)	0.398
750 ARA, mm^2^	0.005 (<0.001, 14.590)	0.193
750 TISA, mm^2^	0.006 (<0.001, 16.824)	0.205
750 TIA, °	0.972 (0.912, 1.036)	0.387

AOD, angle opening distance; ARA, angle recess area; AS-OCT, anterior segment-optical coherence tomography; cpRNFL, circumpapillary retinal nerve fiber layer; IOP, intraocular pressure; logMAR, logarithm of the minimum angle of resolution; mGC/IPL, macular ganglion cell/inner plexiform layer; POAG, primary open-angle glaucoma; TIA, trabecular–iris angle; TISA, trabecular–iris space area; TM, trabecular meshwork.

## Data Availability

The data that support the findings of this study are not publicly available because they contain information that could compromise the privacy of research participants, but are available from the corresponding author (S.M.) upon reasonable request.

## Supplementary Materials

The following supporting information can be downloaded at: https://www.mdpi.com/article/10.3390/jcm15093341/s1, Table S1. Multivariable analysis of factors associated with surgical failure.

## Abbreviations

The following abbreviations are used in this manuscript:

TM	Trabecular meshwork
SC	Schlemm’s canal
MIGS	Minimally invasive glaucoma surgery
ARA	Angle recess area
AOD	Angle opening distance
TIA	Trabecular–iris angle
IOP	Intraocular pressure
TISA	Trabecular–iris space area
AS SS-OCT	Anterior segment swept-source optical coherence tomography
RNFL	Retinal nerve fiber layer
OR	Odds ratio
CI	Confidence interval
