# Supplementary material for: Trabecular Meshwork Thickness Measured by Swept-Source AS-OCT as a Predictor of Surgical Outcomes After Trabecular Micro-Bypass Stent Implantation
Source: J Clin Med. 2026 Apr 27;15(9):3341. doi: 10.3390/jcm15093341 (PMC13163414; doi:10.3390/jcm15093341)
Supplement: Supplementary file 1 [file jcm-15-03341-s001.zip › jcm-4270551-supplementary.pdf]

Supplementary Table S1. Multivariable analysis of factors associated with surgical failure.

	Multivariate models[1), 2), 3)]	
Parameters	OR (95% CI)	P-value
Sex (reference male)		
Age		
Glaucoma type (reference POAG)		
Axial length		
Spherical equivalent		
Preoperative visual acuity (logMAR)		
Preoperative cpRNFL thickness		
Preoperative mGC/IPL thickness		
Preoperative Visual Field Index		
Preoperative mean deviation		
Preoperative IOP at AS-OCT test		
Angle parameters using AS-OCT		
Mean TM thickness, per 10 µm	2.772 (1.120, 6.860)	0.027[1)]
Nasal TM thickness, per 10 µm	1.642 (1.038, 2.597)	0.034[2)]
500 AOD, mm		
500 ARA, mm^2		
500 TISA, mm^2		
500 TIA, °		
750 AOD, mm		
750 ARA, mm^2		
750 TISA, mm^2		
750 TIA, °		
Temp TM thickness, per 10 µm	3.728 (0.859, 16.179)	0.079[3]
500 AOD, mm		
500 ARA, mm^2		
500 TISA, mm^2		
500 TIA, °		
750 AOD, mm		
750 ARA, mm^2		
750 TISA, mm^2		
750 TIA, °		

Multivariate model 1 adjusted for axial length, preoperative IOP, and mean TM thickness. Multivariate model 2 adjusted for axial length, preoperative IOP, and nasal TM thickness. Multivariate model 3 adjusted for axial length, preoperative IOP, and temporal TM thickness.
